# Relationship between Mother’s emotional intelligence, negative parenting behaviour, Preschooler’s attachment instability, and smart device overdependence

**DOI:** 10.1186/s12889-022-13171-3

**Published:** 2022-04-14

**Authors:** Gumhee Lee, Sungjae Kim

**Affiliations:** 1 College of Nursing, Seoul National University, Seoul, Republic of Korea; 2 College of Nursing, The Research Institute of Nursing Science, Seoul National University, 103 Daehak-ro, Jongno-gu, Seoul, 03080 Republic of Korea

**Keywords:** Smartphone, Behavior, addictive, Child, preschool, Emotional intelligence, Parenting, Object attachment

## Abstract

**Background:**

As smart device overdependence among preschoolers could adversely affect their overall development, it is essential to understand the related factors of such overdependence. Mothers have a large influence on preschoolers; however,, the relationship between mothers’ emotional intelligence, negative parenting behaviour, preschoolers’ attachment instability, and smart device overdependence remain unclear. This study aims to develop and test a structural model to explain smart device overdependence among preschoolers.

**Methods:**

The study collects and analyses data from January to May 2021 from 283 mothers raising children aged 3–6 years in South Korea. Questionnaires regarding mothers’ emotional intelligence and negative parenting behaviour, as well as preschoolers’ attachment instability and smart device overdependence, were used to collect data. The data were analysed by SPSS23.0 and AMOS 23.0 software and a structural equation model was constructed; *p* ≤ 0.05 was taken as significant.

**Results:**

Mothers’ emotional intelligence had a direct negative relationship with mothers’ negative parenting behaviour (β = − 0.44) and an indirect negative relationship with preschoolers’ attachment instability (β = − 0.25) and preschoolers’ smart device overdependence (β = − 0.24). Mothers’ negative parenting behaviour had a direct positive relationship with preschoolers’ attachment instability (β = 0.56) and both direct and indirect positive relationships with preschoolers’ smart device overdependence (β = 0.55). Preschoolers’ attachment instability had a direct positive relationship with preschoolers’s smart device overdependence (β = 0.46).

**Conclusions:**

Mothers’ emotional intelligence, negative parenting behaviour, and preschoolers’ attachment instability are associated with preschoolers’ vulnerability to smart device overdependence. These results are shown that more attention is needed to these variables in order to reduce preschoolers’ overdependence on smart devices. Additionally, we propose to develop and provide interventions based on these results.

## Background

### Rationale for the study

Advances in information and communication technology (ICT) have popularised the use of smart devices worldwide, and young children are accordingly being exposed to smart devices such as smartphones, tablet computers, and smart televisions from an early age [1–4]. Notably, the great majority (96.6%) of infants aged 6 months or older are shown music or other videos on a smart device by their parents [2, 3]. Further, young children aged 2 years or older use a smart device nearly every day, and they develop the skills to manipulate a smart device on their own by age 3 or 4 [2].

The main reason for the introduction of smart devices to young children is the benefits of such devices. For example, smart devices are useful tools for education [5] and for other indoor activities—responding to the extended time spent indoors during the coronavirus disease 2019 (COVID-19) pandemic [4]. Further, they provide entertainment that can engage young children and enable parents to control their children better in public spaces [2, 5].

Despite the usefulness of smart devices for rearing children in modern society, the excessive use of smart devices has several adverse effects on the growth and development of young children. For instance, prolonged repetitive use impairs visual acuity [6], shortens sleep duration [7], and threatens physical health by causing musculoskeletal deformities [8]. Furthermore, the psychological repercussions of smart device overdependence include emotional and behavioural problems such as depression, anxiety, somatic complaints, attention problems, and aggressive behaviours [9]. Unlike bidirectional stimulation from one’s caregiver, unidirectional stimulation from a smart device increases the risk of social withdrawal [9]. Therefore, although smart devices can be beneficial in some respects, the excessive use thereof can take a toll on the overall development of young children and thus calls for caution.

Smart device overdependence refers to a state in which excessive use of smart devices increases the prominence and reduces the ability to control their use, resulting in problematic outcomes [10]. It is important to identify the triggers of smart device overdependence in young children and to consider its adverse impacts on them. As early childhood marks the beginning of learning self-control skills, young children still lack the ability to adequately control their own use of smart devices [11, 12]. In addition, it has been reported that the parents play a central role. Since mothers are frequently primary caregivers, the effect size of their associated correlation coefficient is more significant than that of fathers [12]. Thus, maternal factors that may impact young children’s smart device overdependence have important implications.

Since young children’s use of smart devices has increased rapidly within the past 10 years [1–4, 6, 13], it is still difficult to find a mid-range theory to explain young children’s overdependence on smart devices. Due to childbirth and parenting, mothers of young children are prone to not only stress, but also negative emotions such as depression, anxiety, frustration [14–16]. But uncontrolled negative emotions can relate to not only the mother’s own overdependence on smart devices, a variable strongly associated with young children’s overdependence on smart devices [14–17], but also directly to overdependence on smart devices in young children [12]. This suggests that mothers’ ability to regulate various negative emotions is associated with young children’s overdependence on smart devices. In this study, emotional intelligence was selected as a variable to explain the mother’s overall ability to recognize and control various negative emotions [18].

Emotional intelligence is defined as the ability to rationally process and control emotions [18]. It is reported that mothers with high emotional intelligence provide their children with positive parenting behaviours in daily life [19], which means that they limit undesirable behaviours while expressing emotional warmth by controlling their negative emotions [20]. Mothers who demonstrate positive parenting behaviours limit their young children’s overuse of smart devices through these behaviours [12]. Conversely, if mothers cannot control their negative emotions, it can hard to control internalized problems such as anxiety, and the influence of negative emotions can lead to negative parenting behaviours such as neglecting children, leading to children’s smart device overdependence [21]. Meanwhile, attachment refers to the intimate bond between a child and their primary caregiver [22]; to achieve attachment stability, mothers have to pay close attention to and respond to the needs of their young children [23]. However, mothers with low emotional intelligence cannot sensitively identify young children’s needs and respond appropriately [19]; consequently, the likelihood of building attachment stability based on emotional sensitivity declines [23]. Furthermore, young children who experience psychological instability due to failure to achieve attachment stability are reported to be more vulnerable to becoming overuse with smart devices in order to attain psychological safety [24]. This suggests that mothers’ low emotional intelligence would be positively associated with young children’s smart device overdependence through the mediation of mother’s negative parenting behaviour and preschooler’s attachment instability.

Studies have reported that parenting competency–related factors, such as mothers’ smart device overdependence, parenting stress, parenting self-efficacy, parenting behaviours, and parenting attitudes, correlate with smart device overdependence in young children [12]. Moreover, they report that these relationships are mediated by mothers’ parenting behaviours [25], young child–maternal communication [26], young children’s attachment stability [27], and young children’s self-regulation [28]. However, few studies have investigated the direct and indirect relationships of mothers’ emotional intelligence with the smart device overdependence of young children, though it is a fundamental factor thereof [12, 25, 28, 29]. Moreover, studies that have presented an overall model of the relationships among mothers’ emotional intelligence, parenting behaviours, young children’s attachment instability, and young children’s smart device overdependence as an outcome measure are rare [25–28].

It is important to improve mothers’ perception with smart devices in order to prevent overdependence on smart devices in young children [30]. However, changes in the perception of risk factors can cause emotional changes, such as the development of anxiety [31]. If mothers’ emotional intelligence can be related to young children’s overdependence on smart devices, it is necessary to think about how to set the scope of interventions for them.

In this context, this study investigates the effects of mothers’ emotional intelligence on their young children’s smart device overdependence in Korea. Further, develop and empirically test a hypothetical model that describes the mediation of this relationship by parenting behaviours and attachment instability. This study will provide many clues to reducing young children’s overdependence on smart devices.

### Objectives

This study aims to develop and empirically test a multidimensional smart device overdependence model for young children. The specific objectives are as follows: first, to develop and evaluate the fit of a hypothetical model that describes the relationships among factors associated with smart device overdependence in young children; and second, to investigate the relationships among the predictors of smart device overdependence and their effects on smart device overdependence in young children.

### Conceptual framework and hypothetical model

We create a hypothetical model by establishing paths among the study parameters based on previous findings. For example, previous studies have reported that mothers’ emotional intelligence is related to their negative parenting behaviours [18, 19] and young children’s attachment instability [23]. Further, they report that negative parenting behaviours are associated with attachment instability [32, 33]; and that negative parenting behaviours [12, 25] and attachment instability [12, 24] are related to young children’s smart device overdependence. Thus, we can infer that mothers’ emotional intelligence is related to young children’s smart device overdependence [12, 18, 19, 24].

**Fig. 1:**
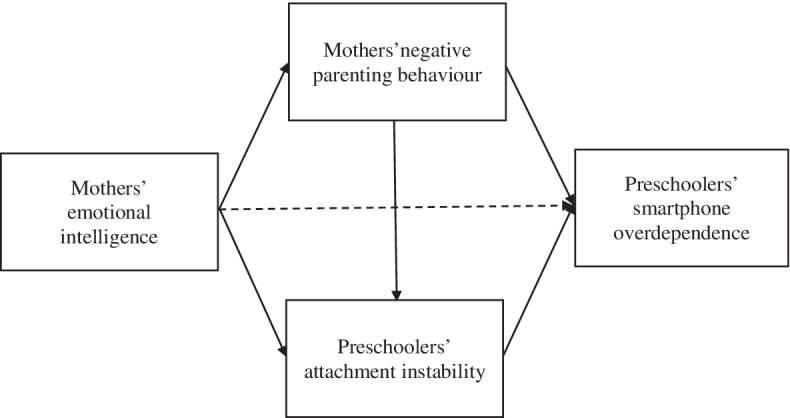
Conceptual framework of this study

Hence, the conceptual framework of this study is established: Young children’s attachment instability is associated with mothers’ emotional intelligence and negative parenting behaviours and ultimately influences young children’s smart device overdependence (Fig. 1). In the hypothetical model, the exogenous variable is the mothers’ emotional intelligence. The endogenous variables are the mothers’ negative parenting behaviours, young children’s attachment instability, and smart device overdependence. Thus, mothers’ negative parenting behaviours mediate the relationship between emotional intelligence and young children’s smart device overdependence. Further, young children’s attachment instability is the mediator in the relationship between negative parenting behaviours and young children’s smart device overdependence (Fig. 1).

## Methods

### Participants

Although mothers of young children aged 0–6 years old are the target study population, we limited the sample to mothers of preschoolers aged 3–6 years old. This is because most instruments assessing smart device usage available in Korea are developed for young children aged 3–6 years [10]. Further, a structural equation model requires an adequately large and reasonable sample size. The minimum sample size for maximum likelihood estimation (MLE) is 200, and a sample size of 200–400 is considered adequate [34]. To recruit participants, we posted an announcement on online communities for mothers in Korea. As a result, 336 mothers voluntarily participated in the online survey. We excluded data with careless responses, resulting in 283 participants being included in the analysis.

### Instruments

#### General characteristics

The following general characteristics were investigated: sex, age, education level, employment status, religion, monthly household income, and preschoolers’ and mothers’ usage time of smart devices.

#### Emotional intelligence

We use the adult emotional quotient test adapted and used by Moon (1996) [35, 36] based on the emotional intelligence model of Salovey and Mayer (1990) [18]. This tool comprises 45 items in five domains: emotional recognition (8 items), emotional expression (7 items), emotional engagement (7 items), emotional control (15 items), and emotional utilisation (8 items) [35, 36]. The total score ranges from 45 to 127, with a higher score indicating higher emotional intelligence. The Cronbach’s α was 0.87 at the time of development [35] and 0.84 in this study.

#### Negative maternal parenting behaviours

We use the maternal parenting behaviours questionnaire developed by Park and Lee [20] and modified and adapted by Choi (2008) [37]. We utilise 10 items for negative parenting behaviours, four for overprotection and permissibility, and six for rejection and neglect. Each item is rated on a 5-point scale Likert-type from 1 (strongly disagree) to 5 (strongly agree), with a higher score indicating more frequent engagement in a particular parenting behaviour by a mother. In a previous study [37], Cronbach’s α of the negative parenting behaviour scale was 0.63–0.83, and in this study, 0.69.

#### Preschoolers’ attachment instability

We utilise the mother–child attachment stability developed by Waters and Deane (1985) and modified and adapted by Lee (2009) [38]. This tool evaluates the stability of children’s attachment to their mothers based on the mothers’ observation of their children using a 4-point Likert scale ranging from 1 (strongly disagree) to 4 (strongly agree). The tool comprises 12 items for attachment stability and 12 items for attachment instability. We used the 12 items for attachment instability in this study. The Cronbach’s α was 0.74 in a previous study [38] and 0.83 in this study.

#### Preschoolers’ smart device overdependence

We use the smartphone overdependence scale for children (ages 3–9) developed by the National Information Society Agency [10]. This 9-item tool comprises three items for the failure of control, three items for salience, and three items for problematic use. Each item is rated on a 4-point Likert scale ranging from 1 (strongly disagree) to 4 (strongly agree). For example, a score of 24–27 indicates a potential risk of smart device overdependence, and a score of 28 or higher indicates a high risk thereof. The Cronbach’s α was 0.75–0.83 at the time of development. In this study, the Cronbach’s α is 0.80 for salience, 0.78 for the failure of control, and 0.80 for problematic use. The overall Cronbach’s α is 0.87. We obtained the developers’ permission for all instruments used in this study.

### Data collection

Data were collected between January and May 2021. In this study, participants were recruited using three Korean web sites (Bunta, Daejeon Mom Cafe, and Moms Holic) that constitute representative online communities used by mothers raising young children in Korea and that allowed us to publicize this study. These online communities are operated for the purpose of providing childcare information. In order to recruit participants, an official promotional statement was posted on the online bulletin boards in these communities. Participants voluntarily clicked on the questionnaire link and read the study information page or contacted the researcher; only those who consented to participate proceeded with the online survey.

### Ethical considerations

The study was conducted with the approval of the institutional review board (IRB) of S National University (IRB no. 2012/002–014). The information sheet provided to potential participants explained the purpose of the study, the questionnaire process, the anonymous processing of written consent forms, confidentiality, and the freedom to withdraw at any time without any disadvantages. Moreover, the participants were informed that unnecessary personal information (e.g., names and addresses) would not be collected and that the collected data would be given identification codes for statistical analysis. The collected data were stored in password-protected files and were only accessed by the principal investigator to protect the participants’ personal information.

### Data analysis

The collected data are analysed using SPSS 23.0 and AMOS 23.0. The participants’ general characteristics are analysed using descriptive statistics, and the reliability of the study is analysed using Cronbach’s α. The normality of the sample was tested with mean, standard deviation, skewness, and kurtosis using multivariate normality tests, and the correlations among the measurement variables are analysed using Pearson’s correlation coefficients. The model parameters are estimated via MLE, and the model fit is assessed using the following: absolute fit indices χ^2^, χ^2^/df, standardised root mean residual (SRMR), and root mean square error of approximation (RMSEA); incremental fit indices comparative fit index (CFI), normed fit index (NFI), and Tucker–Lewis index (TLI); and parsimonious fit index Akaike information criterion (AIC). The specific analysis methods are as follows: The significance of model paths was analysed with regression coefficient, standard error, critical ratio (CR), and *p* value, and the explanatory power of the endogenous variables is analysed with squared multiple correlations. The significance of the direct, indirect, and total effects is analysed with bootstrapping.

## Results

### General characteristics

Table 1 describes the characteristics of the 283 participants in this study. In all, 74 out of 283 (26.1%) preschoolers are considered at risk of smart device overdependence, which is slightly higher than the 25.7% according to a 2020 National Information Society Agency report [10].

**Table 1:** General characteristics (*N* = 283)

Variable	Category	n	%	M ± SD
Mother’s age (years)	< 35	93	32.9	36.3 ± 3.9
	36–40	150	53.0	
	41<	40	14.1	
Mother’s education	< High school graduate	8	2.8	
	Vocational college	35	12.4	
	College graduate	186	65.7	
	Over graduate school	54	19.1	
Mother’s occupation	Full-time	167	59.0	
	Not employed (including leave)	85	30.0	
	Part-time	31	11.0	
Mother’s smart device usage time (daily)	≤0.5 h	47	16.6	2.0 ± 2.0
	< 0.5–1 h	78	27.6	
	< 1–2 h	75	26.5	
	< 2–3 h	36	12.7	
	3 h<	47	16.6	
Household monthly income (Korean 10 thousands won^a^)	< 200	1	0.4	
	201–300	29	10.3	
	301–400	49	17.3	
	401–500	57	20.1	
	501<	147	51.9	
Preschooler’s gender	Boys	130	45.9	
	Girls	153	54.1	
Preschooler’s age (years)	3	61	21.6	4.3 ± .9
	4	118	41.7	
	5	73	25.8	
	6	31	10.9	
Preschooler’s birth order	First	201	71.0	
	Second	69	24.4	
	Third<	13	4.6	
Purpose of use (multiple responses, *n* = 474)	Education	158	33.3	
	Recreation	156	32.9	
	Babysitting	70	14.8	
	Begging	64	13.5	
	Others	26	5.5	
Preschooler’s smart device usage time (daily)	≤0.5 h	125	44.2	1.1 ± 1.5
	< 0.5–1 h	63	22.3	
	< 1–2 h	66	23.3	
	< 2–3 h	12	4.2	
	3<	17	6.0	
Preschooler’s smartphone usage level	< 24 (normal usage group)	209	73.9	19.1 ± 6.3
	24–27 (risk of overdependence group)	57	20.1	
	28 ≤ (overdependent group)	17	6.0	

^a^8.39 United States Dollar

### Descriptive statistics and correlational analysis of study variables

To test for multivariate normality—a requirement for statistical analysis using structural modelling—we compute the skewness and kurtosis values for the measurement variables [34]. The skewness values range from − 0.279 to 0.419, and kurtosis values range from − 0.998 to − 0.228; both satisfy the conditions for normality (Table 2).

**Table 2:** Descriptive statistics and normality

Variable	Range	Total M ± SD	Mean of item M ± SD	Skewness	Kurtosis
Mothers’ emotional intelligence	1–3	95.1 ± 10.1	2.1 ± 0.2	−0.279	− 0.447
Mothers’ negative parenting behaviour	1–5	26.9 ± 5.7	2.7 ± 0.6	0.201	−0.228
Preschoolers’ attachment instability	1–4	24.7 ± 6.5	2.1 ± 0.5	0.369	−0.497
Self-control failure	1–4	6.7 ± 2.5	2.2 ± 0.8	0.118	−0.972
Salience	1–4	6.8 ± 2.6	2.3 ± 0.9	0.233	−0.900
Serious consequences	1–4	5.7 ± 2.4	1.9 ± 0.8	0.419	−0.998
Preschoolers’ smart device overdependence	1–4	19.1 ± 6.3	2.1 ± 0.7	0.059	−0.819

In the bivariate correlation analysis, mothers’ emotional intelligence is found to be significantly negatively correlated with mothers’ negative parenting behaviours (*r* = − 0.437, *p* < 0.001) and preschoolers’ attachment instability (*r* = − 0.225, *p* < 0.001) and negatively with preschoolers’ smart device overdependence (*r* = − 0.143, *p =* 0.016). Mothers’ parenting behaviours are positively correlated with preschoolers’ attachment instability (*r* = 0.563, *p* < 0.001), preschoolers’ smart device overdependence (*r* = 0.508, *p* < 0.001), and all the sub-domains of smart device overdependence (*r* = 0.349–0.501, *p* < 0.001 for all). Preschoolers’ attachment instability is positively correlated with preschoolers’ smart device overdependence (*r* = 0.547, *p* < 0.001), and all sub-domains of smart device overdependence (*r* = 0.386–0.591, *p* < 0.001 for all).

To increase the validity of a model, exogenous variables must be controlled based on theoretical evidence, and control variables should be correlated with the dependent and independent variables [39]. This study eliminates the confounding effect of exogenous variables, based on previous studies in which demographic factors such as mother’s employment status, age, educational level, and house income have had little impact on smart device overdependence in preschoolers [12] and mothers [17]. Furthermore, the bivariate analysis results show that the mothers’ usage time of smart devices were not significantly associated with the independent variables (Table 3).

**Table 3:** Pearson’s correlation among study variables

Variable	1	2	3	4	5	6	7
1. Mothers’ emotional intelligence	**–**						
2. Mothers’ negative parenting behaviour	−0.437^***^	–					
3. Preschoolers’ attachment instability	−0.225^***^	0.563^***^	–				
4. Self-control failure	−0.110	0.349^***^	0.390^***^	–			
5. Salience	−0.047	0.414^***^	0.386^***^	0.414^***^	–		
6. Serious consequences	−0.205^**^	0.501^***^	0.591^***^	0.495^***^	0.683^***^	–	
7. Preschoolers’ smart device overdependence	−0.143^*^	0.508^***^	0.547^***^	0.764^***^	0.850^***^	0.872^***^	
8. Mothers’ smart device usage time	−0.104	0.033	0.002	−0.180^**^	−0.041	− 0.096	−0.129^*^

^***^p **< 0.001,** ^**^p **< 0.01,** ^*^ ***p*** **< 0.05**

### Evaluation of the structural model

#### Hypothetical model

**Fig. 2:**
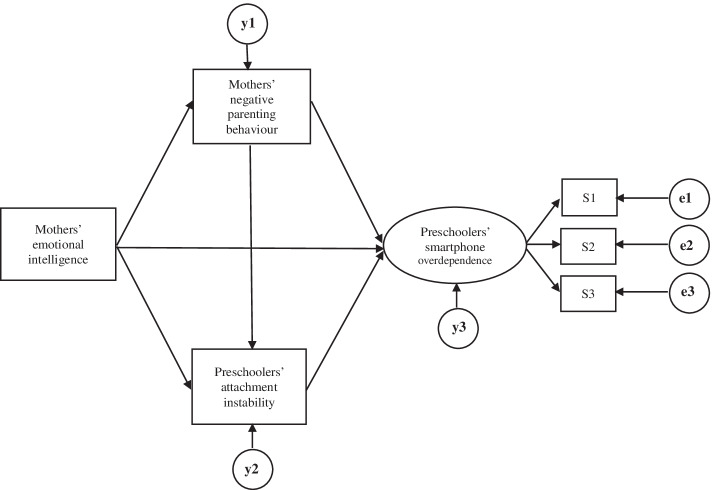
Hypothetical model. S1 = self-control failure; S2 = salience; S3 = serious consequences

The hypothetical model is deemed fit based on the overall fit indices: χ^2^ = 21.245 (DF = 6, *p* = 0.002), χ2/df = 3.541, CFI = 0.974 (≥0.9), NFI = 0.964 (≥0.9), SRMR = 0.031 (≤0.1), RMSEA = 0.095(0.053–0.14) (< 0.1), AIC = 51.245. However, in the path analysis, the paths from emotional intelligence to smart devices and emotional intelligence to attachment instability are insignificant (Fig. 2).

#### Final model and analysis

**Fig. 3:**
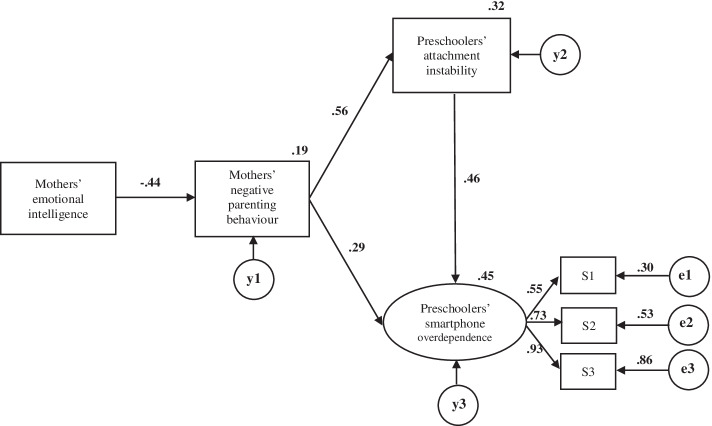
Path diagram for the modified model. S1 = self-control failure; S2 = salience; S3 = serious consequences

The final model confirmed the absence of common method bias (*p* = 0.678) and eliminated paths with absolute CRs lower than 1.96 to increase the simplicity of the model. Thus, the paths from emotional intelligence to attachment instability (t = − 0.472, *p* = 0.637) and emotional intelligence to smart device overdependence (t = − 0.764, *p* = .445) were removed. The final model has a better fit: χ^2^ = 22.023, DF = 8, *p* = 0.005, χ2/df = 2.753, CFI = .976 (≥0.9), NFI = .963 (≥0.9), SRMR = .0366 (≤0.1), RMSEA = 0.079(0.040–0.12) (< 0.1), AIC = 48.023 (Fig. 3).

In the final model, four paths are found to have significant standardised path coefficients. Preschoolers’ smart device overdependence is significantly related with mothers’ negative parenting behaviours (β = 0.29, *p* < 0.001) and preschoolers’ attachment instability (β = 0.46, *p* < 0.001) (Fig. 3, Table 4). Regarding the indirect correlation within the model, the mothers’ negative parenting indirectly related with the preschoolers’ smart device overdependence through the mediation of the preschoolers’ attachment instability behaviours (β = 0.26, *p* < 0.001). Emotional intelligence negatively related with preschoolers’ smart device overdependence through the mediation of parenting behaviours (β = − 0.13, *p* < 0.001). Mothers’ emotional intelligence also has a significant indirect related with on preschoolers’ smart device overdependence through the sequential mediation of negative parenting behaviours and preschoolers’ attachment instability (β = − 0.11, *p* < 0.001) Therefore, the indirect relationship between mothers’ emotional intelligence and preschool children’s dependence on smart devices was confirmed to be β = − 0.24 (*p* < 0.001) in total (Table 4).

**Table 4:** Parameter estimation and statistical significance testing for the structural model

Path analysis	Direct effect β (***p***)	Indirect effect β (***p***)	Total effect β (***p***)	SMC
Mothers’ emotional intelligence → Mothers’ negative parenting behaviours	−.44^***^		−.44^***^	**.191**
Mothers’ emotional intelligence → Preschoolers’ attachment instability		−.25^***^	−.25^***^	**.317**
Mothers’ emotional intelligence → Preschoolers’ smart device overdependence		−.24^***^	−.24^***^	**.446**
Mothers’ negative parenting behaviour → Preschoolers’ attachment instability	.56^***^		.56^***^	
Mothers’ negative parenting behaviour → Preschoolers’ smart device overdependence	.29^***^	.26^***^	.55^***^	
Preschoolers’ attachment instability → Preschoolers’ smart device overdependence	.46^***^		.46^***^	

*SMC* = squared multiple correlations. ^***^p **< 0.001**

## Discussion

This study establishes a model that explains the relationship between mothers’ emotional intelligence, negative parenting behaviours, preschoolers’ attachment instability, and preschoolers’ smart device overdependence based on scientific evidence and identifies the relationships among the variables. We now discuss these relationships among the factors, namely the direct relationship of preschoolers’ attachment instability and mothers’ negative parenting behaviours and the indirect relationship of mothers’ emotional intelligence, on smart device overdependence among preschoolers.

In our study, the attachment instability of preschoolers is found to have the strongest relationship with their smart device overdependence. This suggests that preschoolers who develop attachment instability fail to obtain psychological safety from their mothers and thus seek psychological safety using smart devices. Previous arguments that young children may become overdependent on smart devices as they experience psychological safety from the firm touch of such devices and find entertainment in the repeated use of the same support our findings [40]. Developing attachment stability is important in young children because attachment is essential to the survival of young children, and mothers’ response serves as a social mirror that shapes the formation of young children’s egos [22]. This differs from the importance of peer relationships or school adjustment. It is considered in the evaluation of smart device overdependence in school-age children and adolescents [41, 42] because they compare themselves with their peers and place great emphasis on other people’s judgments [43]. The findings of this study reflect the developmental features of young children.

Mothers’ negative parenting behaviours likewise was directly related to preschoolers’ smart device overdependence. Negative parenting behaviours include rejecting, uninvolved, overprotective, and uncritical/permissive parenting styles [37]. This study shows that contrary to the belief that a rejecting parenting style, such as getting angry or yelling, would reduce smart device overdependence among young children, it increases smart device overdependence. This is supported by the argument that negative parenting behaviours, such as immediately expressing anger toward young children’s negative behaviours, reinforces the children’s problematic behaviours through a secondary benefit—attention [44]. Moreover, we can infer that uninvolved, overprotective, and uncritical/permissive parenting behaviours, in which mothers do not properly restrict young children’s use of smart devices also increases the risk of smart device overdependence. This is because inattention and the repeated use of smart devices to control young children increase their smart device overdependence [12] while failing to meet their actual needs, resulting in unmet needs, and increasing attachment instability [12, 23, 33] and thereby increasing the use of smart devices.

Meanwhile, the finding that preschoolers’ attachment instability has a stronger correlation with smart device overdependence than mothers’ negative parenting behaviours suggests that smart device overdependence in preschoolers is not a simple behaviour problem that can be resolved by restricting usage. Instead, it is a psychological issue that requires evaluating and reducing attachment instability. This result provides practical implications for interventions that target the reduction of smart device overdependence in young children. In other words, such interventions should simultaneously aim to improve mothers’ negative parenting behaviours and improve young children’s attachment instability. While parents should restrict young children’s excessive use of smart devices, parents should also avoid negative parenting styles, such as criticising and stigmatising, that may hinder their young children’s development of attachment stability. This is in line with the argument that positive behaviours should be properly rewarded with compliments and acceptance to modify young children’s problem behaviours [44], while conversely, young children’s needs should be assessed, and attachment instability should be improved based on emotional attentiveness [23]. This also supports the argument that young children’s problem behaviours caused by ignorance, boredom, and frustration should be corrected by simply teaching them or changing their direction of interest [44]. Hence, even if young children’s use of smart devices cannot be completely avoided given the current challenges in engaging in outdoor activities, it is necessary to enforce proper limits. These may include setting an allowed screen time [12]. In the future, interventions that ameliorate mothers’ parenting behaviours and reduce young children’s attachment instability should be offered.

Other activities that can replace smart devices should be developed. Thus, based on our findings, we recommend attachment-building play. Play is a useful means to improve attachment instability between a caregiver and young children [45]. Even if negative parenting behaviours have damaged their attachment, both the caregivers and young children are in an equal position during play and participate in compliance with democratic rules of play. Moreover, it does not necessarily require professional tools [46]. Thus, based on our findings, we suggest further studies to develop and evaluate the effects of interventions consisting of attachment-building play on smart device overdependence in young children.

Our study shows that mothers’ emotional intelligence was not directly associated with preschoolers’ smart device overdependence but was instead directly related to mothers’ negative parenting behaviours and indirectly to preschoolers’ attachment instability. This shows that it is essential to boost emotional intelligence in mothers, that is, the ability to identify and control one’s own emotions to lower their negative parenting behaviours and preschooler’s attachment instability. In addition, the mother’s emotional sensitivity and responsiveness are emphasized as important factors to reduce attachment instability [23], and in order to increase the mother’s emotional sensitivity and responsiveness, it is desirable to increase the mother’s emotional intelligence [47]. This will contribute to a long-term, lasting reduction of young children’s dependence on smart devices. However, emotional intelligence can only be improved with a thorough understanding of emotional intelligence and training to enhance emotional sensitivity [47], and not simply by ‘converting’ negative emotions [48]. Thus, it is necessary to provide interventions that improve maternal emotional intelligence based on a deep understanding of emotional intelligence.

Meanwhile, in order to reduce young children’s overdependence on smart devices, it is also important to improve the related perceptions and knowledge of mothers [30]. However, the results of this study confirming that mothers’ low emotional intelligence is related to overdependence on smart devices in young children provide important implications. Increased perception and knowledge of threats can cause anxiety [31], and this may perception of the young child’s overdependence on smart devices or knowledge of the harmful effects caused by overdependence. However, mothers with low emotional intelligence and poor communication skills cannot effectively control negative emotions, which can lead to negative parenting behaviours such as yelling, threatening, or stigmatizing. This discussion is in line with the argument that anxious mothers impose more punishment on their young children [49]. Based on this study, these negative parenting behaviours can lower young children’s attachment to their mother and increase overdependence on smart devices in young children, and mothers who need more attention due to low emotional intelligence and their children can suffer more than others. These discussions suggest that interventions to improve perception and knowledge about smart device overdependence should be systematically prepared by carefully considering emotions and variables related to smart device overdependence. The results of this study are evidence of the importance of emotions with regard to overdependence on smart devices.

Based on this study, even if the ultimate goal is to reduce young children’s smart device overdependence, it is necessary to assess and improve mothers’ emotional intelligence and negative parenting behaviours as independent variables. In addition, assessing and reducing preschoolers’ attachment instability would be an effective way to foster decrease in preschoolers’ smart device overdependence in the long term.

Mothers’ smart device usage time was negatively correlated with young children’s smart device overdependence, contradicting previous findings [12]. Possibly, the higher percentage of working mothers as compared to stay-at-home mothers in our study and the consequent increase in household income may have led to more participation in other activities instead of using a smart device. Additionally, as many mothers worked at home to reduce the risk of contracting COVID-19, their use of a smart device would have been primarily work-related [50]. This would have led to a difference in young children’s social learning as they observed their mother using a smart device [12].

This study has several limitations. First, we did not survey reasons for mothers’ smart device use, so we could not examine the effects of such reasons on young children’s smart device overdependence. Second, we could not examine the effects of the subfactors of each parameter; consequently, the explanatory power of the sub-variables could not be clearly understood, and there may be a bias in the parameter values [34]. Finally, as the data were only collected in Korea and used a convenience sampling method, the generalizability of the findings may be limited. Therefore, based on the discussion in this study, future studies should ask questions about reasons for using smart devices along with the time mothers use smart devices, and follow-up studies in other countries should be conducted.

Nonetheless, this study sheds light on the specific variables to be considered in smart device overdependence among young children and the direction of interventions needed to reduce the repercussions of smart device overdependence, as the use of smart devices among young children is inevitable in the modern world. In particular, this study has high structural validity for the importance of mothers’ emotional intelligence in smart device overdependence among young children and for the relationships of mothers’ emotional intelligence on young children’s smart device overdependence through the mediation of mothers’ negative parenting behaviours and young children’s attachment instability using structural modelling. The findings of this study will serve as valuable foundational data for parental education and for experts in multiple disciplines who work with parents.

## Conclusions

The model tested in this study was suitable to explain the relationships of mothers’ emotional intelligence on young children’s overdependence on smart devices. Owing to mothers’ large influence on their young children, it is essential to improve mothers’ emotional intelligence and reduce negative parenting behaviours and (thereby) young children’s attachment instability in order to also reduce their overdependence on smart devices. Based on these results, it is suggested that interventions be developed, and their effectiveness evaluated to improve parents’ emotional intelligence and negative parenting behaviors so as to increase young children’s attachment stability and reduce smart device overdependence.

## Data Availability

All data generated or analysed during this study are included in this published article.

## Abbreviations

AIC: Akaike information criterion
CFI: Comparative fit index
CR: Critical ratio
COVID-19: Coronavirus disease 2019
ICT: Information and communication technology
MLE: Maximum likelihood estimation
NFI: Normed fit index
RMSEA: Root–mean–square error of approximation
SRMR: Standardised root–mean residual
TLI: Tucker–Lewis index
